# Global analysis of mRNA stability in the archaeon *Sulfolobus*

**DOI:** 10.1186/gb-2006-7-10-r99

**Published:** 2006-10-26

**Authors:** Anders F Andersson, Magnus Lundgren, Stefan Eriksson, Magnus Rosenlund, Rolf Bernander, Peter Nilsson

**Affiliations:** 1 Department of Gene Technology, School of Biotechnology, KTH - Royal Institute of Technology, AlbaNova University Center, SE-106 91 Stockholm, Sweden; 2 Department of Earth and Planetary Science, University of California, Berkeley, Berkeley, CA 94720-4767, USA; 3 Department of Molecular Evolution, Evolutionary Biology Center, Uppsala University, SE-752 36 Uppsala, Sweden; 4 Department of Mathematics, KTH - Royal Institute of Technology, SE-100 44 Stockholm, Sweden

## Abstract

**Background:**

Transcript half-lives differ between organisms, and between groups of genes within the same organism. The mechanisms underlying these differences are not clear, nor are the biochemical properties that determine the stability of a transcript. To address these issues, genome-wide mRNA decay studies have been conducted in eukaryotes and bacteria. In contrast, relatively little is known about RNA stability in the third domain of life, Archaea. Here, we present a microarray-based analysis of mRNA half-lives in the hyperthermophilic crenarchaea *Sulfolobus solfataricus *and *Sulfolobus acidocaldarius*, constituting the first genome-wide study of RNA decay in archaea.

**Results:**

The two transcriptomes displayed similar half-life distributions, with medians of about five minutes. Growth-related genes, such as those involved in transcription, translation and energy production, were over-represented among unstable transcripts, whereas uncharacterized genes were over-represented among the most stable. Half-life was negatively correlated with transcript abundance and, unlike the situation in other organisms, also negatively correlated with transcript length.

**Conclusion:**

The mRNA half-life distribution of *Sulfolobus *species is similar to those of much faster growing bacteria, contrasting with the earlier observation that median mRNA half-life is proportional to the minimal length of the cell cycle. Instead, short half-lives may be a general feature of prokaryotic transcriptomes, possibly related to the absence of a nucleus and/or more limited post-transcriptional regulatory mechanisms. The pattern of growth-related transcripts being among the least stable in *Sulfolobus *may also indicate that the short half-lives reflect a necessity to rapidly reprogram gene expression upon sudden changes in environmental conditions.

## Abstract 2

A microarray-based analysis of mRNA half-lives in two species of Sulfolobus, an hyperthermophilic archaeon, shows that their mRNA half-life distribution is similar to that of much faster growing bacteria

## Background

Studies of gene regulation have traditionally focused on transcription initiation. However, recent discoveries that altered mRNA stability under some conditions plays an equally important role in the dynamic control of gene expression [1] have emphasized the importance of also taking RNA turnover into account. Also when the stability of a transcript is unchanged there are important consequences for gene regulation since, upon changes in the rate of transcription, the stability of an RNA species determines how fast a new steady-state level will be reached [2]. Moreover, the half-life will influence the stochastic fluctuation in the production rate of the corresponding protein [3].

While mechanisms for RNA degradation in bacteria and eukaryotes have been well studied, less is known about the process in organisms from the third domain of life, the Archaea. By computational analysis of gene-order conservation in several archaeal genomes, a protein complex orthologous to the eukaryotic exosome was predicted [4]. This multisubunit complex consists of RNases, RNA helicases and RNA-binding proteins, in which various RNA classes are degraded in a 3' to 5' fashion. Such a complex was later isolated from *Sulfolobus solfataricus *[5], and the exosome core structure was subsequently determinated [6]. Recently, the *S. solfataricus *exosome was demonstrated to display polyadenylation activity, in addition to degradation of RNA [7].

Early understanding of RNA stability was gained from studies of a limited number of individual transcripts. The emergence of microarray technology has, however, facilitated studies at the transcriptome level. Such studies have been conducted in bacteria [8,9] and eukaryotes [10,11], and have provided important insights, such as a relationship between physiological function and mRNA turnover rate. Still, important questions remains to be answered, for example, why half-lives differ between groups of genes with different physiological functions, and which general features of mRNA molecules determine their half-lives.

Although the half-lives (ranging from two minutes to two hours) of individual transcripts have been determined in a range of archaeal species, including thermophiles [12], methanogens [13,14] and halophiles [15], no comprehensive mRNA decay survey has yet been performed. Here, we present the first genome-wide study of mRNA decay in archaea, focused on the hyperthermophilic acidophiles *S. solfataricus *and *S. acidocaldarius*. The aim was to provide an overview of global mRNA half-life in archaea, generally as well as regarding functional groups of transcripts, and to provide the first large-scale data set for comparison with bacteria and eukaryotes.

## Results

### Genome-wide analysis of mRNA half-life

**Figure 1:**
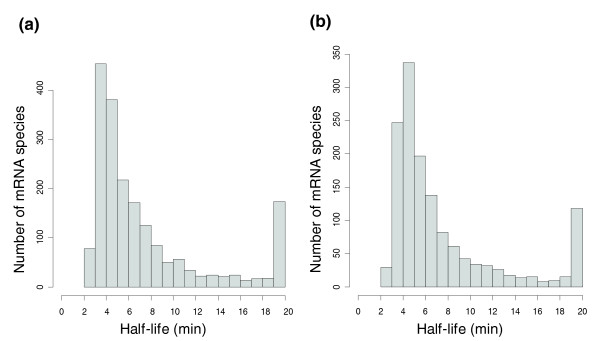
mRNA half-life distribution in **(a) ***S. solfataricus *and **(b) ***S. acidocaldarius*. The right-most bar represents transcripts with t_1/2 _> 19 minutes.

We monitored mRNA decay in exponentially growing cell cultures by measuring transcript abundance at 3, 6, 9, 12 and 15 minutes after transcriptional arrest by actinomycin D, previously demonstrated to block transcription in human cells [16,17], the archaea *Haloferax mediterranei *[15] and *S. solfataricus *[12]. Accurate half-life determinations require rapid and quantitative inhibition, and the analysis is based on the demonstration by Bini *et al*. [12] that these effects are obtained by actinomycin D treatment of *S. solfataricus*. The analysis was focused on the *S. solfataricus *transcriptome, but *S. acidocaldarius *was also surveyed for general trends, providing an independent biological replicate. As we had little *a priori *knowledge on RNA decay rates in these species, we applied a normalization procedure based on the assumption that a given proportion of the transcripts were stable (see Materials and methods). After filtering out genes with missing data points and/or noisy decay profiles, we obtained half-life data for 2,064 and 1,582 genes in *S. solfataricus *and *S. acidocaldarius*, respectively (Additional data file 1). When the proportion of stable transcripts was set to 10%, the median half-life was estimated to 5.3 and 5.1 minutes for *S. solfataricus *and *S. acidocaldarius*, respectively (changing the proportion to 5% did not alter the results significantly; median half-lives 4.4 and 4.5 minutes). Similar half-life distributions were obtained in the two species (Figure 1a, b), with approximately 50% of the transcripts within the 4 to 8 minutes range, and with approximately 8% displaying half-lives longer than 20 minutes. No transcripts with a half-life shorter than 2 minutes were observed. Transcripts with lower stability could potentially be hidden among those with missing data, but there was no bias in missing data points toward the end of the time series that would indicate such a relationship.

**Figure 2:**
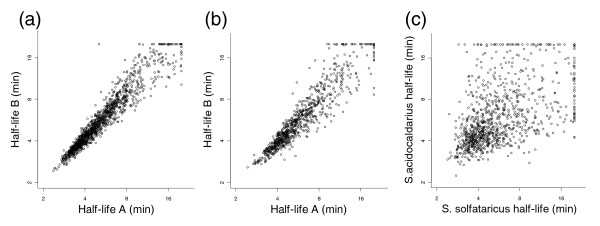
Comparison of mRNA half-lives obtained in **(a) **two independent labeling and hybridization assays, and **(b) **two independent cultures of the same species. **(c) **Comparison of mRNA half-lives of orthologous genes in *S. solfataricus *and *S. acidocaldarius*. Half-lives are shown in log_2_-scale.

The technical reproducibility of the assay was monitored by comparing the half-lives obtained in independent labeling/hybridization series (Figure 2a). The average variation was 10%, as calculated by dividing (absolute) half-life difference with half-life average, and 87% of the transcripts displayed less than 20% variation. By comparing the results obtained in independent cultures, the biological variation could be measured. This was only slightly higher, with 12% average variation and 84% of the transcripts displaying less than 20% variation (Figure 2b). Genes expressed in the same polycistronic messages are expected to display similar half-lives. Few operons have been experimentally verified in *Sulfolobus*, but occurrences of putative transcription initiation sites together with intergenic distance distributions [18,19] indicate that genes on the same strand separated by less than 10 nucleotides are likely to belong to the same operon. We observed a significant correlation (Pearson *r *= 0.72, *P *< 10^-15^) between the half-life of the upstream and downstream gene for such gene pairs in *S. solfataricus *(Additional data file 2).

To confirm the robustness of the data, we performed quantitative real-time PCR (qPCR) on cDNA from all time points for nine transcripts that represented a wide range of half-lives. The half-lives calculated from the qPCR results correlated well (Pearson *r *= 0.94, average half-life variation 17%) with the microarray-derived data (Additional data file 3).

### mRNA half-life in relation to gene function

**Figure 3:**
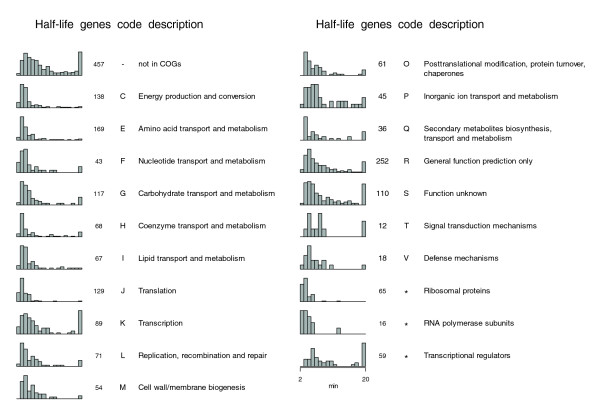
Distributions of *S. solfataricus *mRNA half-lives, and number of genes with estimated half-lives, for different functional categories in the COG database. Only categories with more than nine genes with estimated half-lives are shown. Each bar in the histogram represents the proportion of genes with a half-life within a 1 minute interval, where the first bar includes half-lives of > 2 but ≤ 3 minutes, the second > 3 but ≤ 4 minutes, and so on. The last bar includes all transcripts with half-lives > 19 minutes. The groups 'Ribosomal proteins' and 'RNA polymerase subunits' are based on the annotations in [26] and 'Transcriptional regulators' on annotations in the COG database.

Earlier studies have revealed that transcripts encoding proteins with related functions tend to decay at similar rates [8,10]. To address this issue in *Sulfolobus*, we compared the stability of *S. solfataricus *genes belonging to different functional categories in the COG (clusters of orthologous groups of proteins)database (Figure 3). All genes with measured half-lives were split into four equally sized groups according to half-life, and each group was tested for over-representation of any functional category. Although these categories are rather broad, and include many potential subgroups (for example, anabolic and catabolic genes in the same group), the distributions of half-lives for certain categories deviated significantly from the average. Over-represented categories in the group of shortest half-lives (0 <*t*_1/2 _≤ 4) were 'translation', 'amino acid transport and metabolism' and 'energy production and conversion'. No functional category was significantly over-represented in the other three groups, but genes not present in the COG database (and thus uncommon in other species) were over-represented within the group with longest half-lives (*t*_1/2 _> 9). The most enriched category in the group of longest half-lives was 'inorganic ion transport and metabolism', although not significantly (*P *= 0.14). Although the 'transcription' category was not over-represented in any half-life group, the components of the basal transcription machinery displayed significantly shorter half-lives than average (see below). On the whole, transcripts encoding proteins involved in growth-related processes, such as transcription, tRNA synthesis, translation and central metabolic pathways, generally decayed rapidly, whereas those encoding products necessary for maintaining cellular homeostasis, for example, ion transporters, were more stable. Similar results were obtained in *S. acidocaldarius *(Additional data file 4).

**Figure 4:**
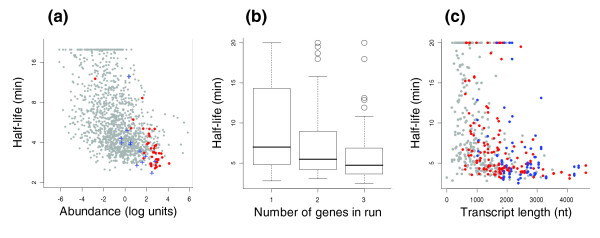
mRNA half-life in relation to transcript abundance, operon size (in number of genes) and transcript length (in nucleotides) in *S. solfataricus*. **(a) **Scatter plot of transcript abundance versus half-life. mRNA half-lives are shown in log_2_-scale. Transcript abundance represents log_2_-transformed expression (cDNA/genomic DNA) ratios, normalized such that the average of the log ratios equals zero. Red and blue dots represent genes for ribosomal proteins and polymerase subunits, respectively. **(b) **Box plots of half-life distributions for transcripts in one-, two- and three-gene runs; 50% of the genes are included in the boxes, and 80% between the whiskers; the line within the box represents the median half-life. **(c) **Scatter plot of mRNA half-life versus transcript length (counting from first start to last stop codon in each run). Genes belonging to one-, two- and three-gene runs are shown in grey, red and blue, respectively.

Many growth-related genes are highly expressed. To investigate the relationship between expression level and half-life further, we measured relative transcript abundance in a sample harvested immediately before actinomycin D was added to the culture. This was done by hybridizing cDNA derived from this culture together with genomic DNA from a stationary phase culture. As all genes are present in equal copy-number in stationary phase cells [20], the cDNA/DNA ratios serve as relative estimates of transcript abundance. Similar to what was demonstrated using the same approach in *Escherichia coli *[8], a negative correlation between transcript abundance and stability was observed in *Sulfolobus *(Spearman rank order *ρ *< -0.4, *P *< 10^-15 ^in both species; Figure 4a and Additional data file 5).

Among the 163 mRNAs in *S. solfataricus *with *t*_1/2 _> 20 minutes, 63% were either not present in the COG database (and lacked functional annotation) or belonged to one of the categories 'function unknown' or 'general function prediction only'. This is significantly more than the 33% in the entire mRNA decay data set, and uncharacterized genes are thus over-represented among those with stable transcripts. Among those with computationally predicted general functions, we found a few groups enriched in stable genes. These included ATPases of the AAA+ superfamily, for which 12 out of 14 transcripts had half-lives above the median, membrane proteins (10 out of 12 above median) and nucleotidyltransferases (7 out of 7). As a final example, within the group of predicted nucleic acid-binding proteins containing a PIN domain, 11 out of 12 transcripts displayed half-lives above median. In *S. solfataricus*, these genes constitute the 'toxin' in the 'toxin-antitoxin' loci of *vapBC*-type that recently were found to be abundant in archaeal species [21]. The cellular target(s) of the VapC toxins is not yet clear, but in eukaryotes PIN domain proteins are ribonucleases involved in nonsense-mediated decay and RNA interference pathways [22], and may thus be involved in related processes in archaeal cells [23,24].

### mRNA stability in regulation of transcription and translation

To investigate the relationship between transcript half-life and protein function in more detail, we focused on the transcriptional and translational apparatuses of *S. solfataricus*. The archaeal transcription machinery is a simplified version of the eukaryal system, in which RNA polymerase, TATA-box-binding protein (Tbp) and transcription factor B (Tfb) are sufficient for initiation from most promoters *in vitro *[25]. Thirteen out of the fourteen subunits of the *S. solfataricus *RNA polymerase [26], as well as Tbp, displayed transcript half-lives below median. Interestingly, the one polymerase subunit that was markedly more stable than the others, RpoG (SSO0277), has only been identified in *Sulfolobus*, indicating a unique function for this protein in an otherwise highly conserved transcriptional apparatus.

Similar to several other archaea, *Sulfolobus *encodes multiple Tfb homologues, and it has been proposed that differential use of these may be a mechanism for gene regulation [27]. Consistent with this hypothesis, one of the *Haloferax volcanii *Tfbs was found to increase in abundance relative to its paralogs in response to heat shock [28]. Interestingly, and in agreement with an earlier study [12], the three transcripts predicted to encode Tfbs in *S. solfataricus *displayed differential half-lives (t_1/2 _= 3.3, 7.8 and 9.1 minutes for SSO0946, SSO0280 and SSO0446, respectively).

In contrast to most transcripts encoding the basal transcription machinery, we found mRNAs encoding transcriptional regulators generally to be long-lived in *S. solfataricus *(Figure 3). Of the 48 genes known or predicted to encode transcriptional regulators, 37 displayed half-lives above median. Some groups, for instance regulators belonging to the Ars family, all showed half-lives above median.

In a growing *S. solfataricus *cell, ribosomal protein mRNAs are among the most abundant (Figure 4a). The regulation of ribosomal protein synthesis, as well as ribosome biogenesis, differs significantly between *E. coli *and budding yeast. Whereas yeast regulation primarily is conducted at the transcriptional level, it mainly occurs at the translational level in *E. coli *[29]. How regulation is conducted in archaea is not known, but since the half-lives of the ribosomal protein transcripts were among the shortest in both transcriptomes, the regulation appears to be conducted at the transcriptional level in *Sulfolobus*.

Two RNAs deviated from the uniform half-life distribution of ribosomal protein transcripts in *S. solfataricus*: *rpl13E *(SSO2442) and *rpl34E *(SSO6374) (t_1/2 _= 12.1 and 8.7 minutes, respectively). The Rpl13E protein belongs to COG4352, which is restricted to eukaryotes and crenarchaea. Interestingly, the gene has been reported to be induced upon cold stress in the plant *Brassica napus *[30], and *E. coli *cells expressing a homologous protein derived from the green algae *Chlamydomonas *were reported to display enhanced tolerance against salt and cold stress [31]. The long transcript half-life in *S. solfataricus *indicates a specialized function for Rpl13E in crenarchaea as well. There are, to our knowledge, no published data indicating a specific role for Rpl34E but COG2174, representing this protein, is absent in several archaeal lineages.

### Half-life in relation to transcript length

Although numerous *cis*-elements and transacting factors affecting half-lives of individual transcripts have been identified in a variety of organisms [32,33], few general determinants of RNA stability are known. Previous genome-wide surveys did not reveal any strong correlations between half-life and GC content, open reading frame length or codon usage [8,10]. Since some rapidly decaying transcripts, encoding, for instance, ribosomal proteins, are expressed as polycistronic messages in *Sulfolobus*, we wished to investigate whether operon length was related to half-life. To find putative operons of defined sizes, we identified contiguous stretches, 'runs', of one, two or three genes encoded on the same strand, separated by less than ten nucleotides, and bounded by genes encoded on the other strand. Half-life was found to decrease with the number of genes in a run (Mann-Whitney test *P *< 0.0001 for one- versus two-gene runs, and *P *< 0.02 for two- versus three-gene runs; Figure 4b). Since this could be a consequence of a correlation between half-life and transcript length (counting in nucleotides) we controlled for this by splitting, based on transcript lengths (counting from first start to last stop codon), all one- and two-gene runs into two sets of equal number of runs. No significant half-life differences were obtained between one- and two-gene runs in either of the two sets (*P *> 0.8 in both). Moreover, half-life was correlated with transcript length within each group of one-, two- or three-gene runs (*P *< 0.02 in all groups; Figure 4c). Hence, transcript length *per se *seemed to be the important factor. The correlation was independent of the correlation between expression level and half-life: transcript abundance and length is not correlated in one-gene runs (*ρ *= 0.01, *P *> 0.6), while in this group half-life is negatively correlated with transcript length (*ρ *= -0.415, *P *< 10^-15^). Similar results were obtained in *S. acidocaldarius *(Additional data file 5).

### Inter-species comparison of mRNA half-lives

Our experimental approach allowed us to compare the mRNA half-lives of orthologous genes in the two *Sulfolobus *species. We observed a highly significant correlation between the log transformed mRNA half-lives of the two organisms (Pearson *r *= 0.51, *P *< 10^-15^; Figure 2c), although many genes displayed differential half-lives. This can be compared with the correlation obtained from different cultures of the same species (Figure 2b; Pearson *r *= 0.93). Thus, although the mRNA half-lives of the two species were correlated, there was substantial deviation that could not be explained by experimental noise.

## Discussion

Previous genome-wide mRNA half-life analyses have indicated that median half-life is roughly proportional to the minimal length of the cell cycle (median half-lives of 5, 20, and 600 minutes correspond to cell cycle lengths of 20, 90, and 3,000 minutes, respectively, for *E. coli*, *Saccharomyces cerevisiae*, and human HepG2/Bud8 cell [8,10,11]). The short transcript half-lives in *Sulfolobus*, with generation times of four to six hours, contrast markedly with this pattern. The similar half-life distributions of the archaeon *Sulfolobus*, the Gram-negative bacterium *E. coli *[8], and the Gram-positive bacterium *Bacillus subtilis *[9] may, alternatively, indicate that short transcript half-lives comprise a general feature of prokaryotic organisms. This could reflect the longer times required for processing and transport out of the nucleus of eukaryotic mRNAs. The added possibilities for post-transcriptional regulation at different stages of processing and transport, and more elaborate mechanisms for controlling translation and transcript stability, may further decrease the need for high mRNA turnover rates. In contrast, *Sulfolobus *transcripts frequently lack untranslated leaders, reducing the potential for translational regulation [12]. Thus, mechanisms for post-transcriptional control may be a more important determining factor for RNA half-life average than cell cycle length and growth rate. This is corroborated by the observation that mRNA half-lives in *E. coli *are similar in nutrient-rich and defined media, in which the generation time is tripled [8].

The short transcript half-lives, with highly expressed genes being among the least stable, may also reflect a necessity to quickly adapt to environmental changes, such as sudden changes in physicochemical conditions or rapid depletion of nutrients, which *Sulfolobus *species are likely to encounter in their natural habitats of geothermal springs. With a high turnover rate, a global, transient, transcriptional arrest would rapidly reduce the level of abundant growth-related transcripts, whereby energy is saved and ribosomes become redistributed to transcripts with lower decay rates, needed for the specific situation.

Recently, stochastic fluctuations in cellular protein levels have been experimentally addressed [34-36], and it appears that high transcription rates in combination with low numbers of translations per mRNA result in relatively low fluctuations in protein levels in both bacteria and eukaryotes. In an analysis of several yeast functional genomic data sets [37], it was found that essential genes, and genes involved in protein complexes, are biased toward adopting this strategy, suggesting that the gene expression machinery is tuned to minimize noise in the expression of proteins for which perturbed concentrations are particularly prone to lower the fitness of the organism. Since high mRNA turnover rates require relatively high transcription rates and allows a limited number of translations per mRNA [3], and since highly expressed genes are enriched in essential functions [38], mechanisms for noise minimization could potentially contribute to the negative correlation between expression level and mRNA longevity observed in *Sulfolobus*.

In a genome-wide study in budding yeast [10], transcripts involved in regulatory systems were suggested to decay rapidly. In contrast, we found many transcriptional regulators to be long-lived in *S. solfataricus*, which indicates that some of these may be controlled at the post-transcriptional level, potentially facilitating faster responses. Moreover, we found basal transcription factors to decay at different rates. This may also have implications for gene regulation: a transient transcriptional arrest could, due to differential half-lives, lead to dramatic changes in relative concentrations of, for instance, Tfbs, which, in turn, may lead to major changes in gene expression patterns.

To our knowledge, no general relationship between transcript length and half-life has been reported previously. It remains to be elucidated if the physical properties of long transcripts make these prone to degradation or, alternatively, if they are enriched in functions for which rapid decay is favorable. Interestingly, in this context, in a genome-wide study of mRNA translation profiles in yeast [39] an inverse correlation was found between ribosome density and open reading frame length, which was observed even within functional subsets of genes.

The mRNA decay was measured in exponentially growing cells, and it cannot be excluded that half-lives may be different in other conditions, for example, in stationary phase cells. Also, it could be argued that actinomycin treatment might induce a stress response. If so, this must have been rapid, since the relative (log) transcript abundances changed linearly over time after three minutes, indicating that new constant rates of decay had been reached for all transcripts already at this early time point.

## Conclusion

We report the first genome-wide study of mRNA decay in archaea. The analysis provides half-life data for thousands of genes of two related species, and will serve as a resource for future analyses of the molecular signatures that determine the stability of RNAs, and provide insights into how transcriptomes are shaped by evolution. We found that, overall, mRNA decay rates are similar to that of bacteria. Global analysis of mRNA decay in additional organisms will reveal if short transcript half-lives comprise a general feature of prokaryotes.

## Materials and methods

### Strains, media and growth conditions

*S. solfataricus *DSM1617 and *S. acidocaldarius *DSM639 (Deutsche Sammlung von Mikroorganismen und Zellkulturen GmbH, Braunschweig, Germany) cultures were grown at 79°C in modified Allen mineral base medium containing 0.2% w/v tryptone [40]. Growth was monitored by optical density (OD) measurements at 600 nm, and doubling times of 3.5 and 6 h were obtained for *S. acidocaldarius *and *S. solfataricus*, respectively (not shown). Flow cytometry analysis was performed as described previously [20], and cell size and DNA content distributions typical for exponentially growing *Sulfolobus *populations were obtained (Additional data file 6). For extraction of RNA, 200 ml cultures were grown in 1 l Erlenmeyer flasks to an OD of 0.1. For extraction of stationary phase genomic DNA, 200 ml cultures were grown in 1 l Erlenmeyer flasks to an OD of 0.7.

### Transcription inhibition by actinomycin D, and sampling

Actinomycin D, dissolved in DMSO, was added to a final concentration of 15 μg/ml and samples (25 ml) were collected for half-life analysis at 3, 6, 9, 12 and 15 minutes after addition, and rapidly chilled on ice by addition of an equal volume of ice-cold medium. Samples were also extracted from untreated cultures to generate reference RNA. At each time point, samples for flow cytometry analysis were also collected and analyzed. No effects were seen on cell and DNA integrity during this time interval (Additional data file 6).

### Labelling of cDNA and genomic DNA

RNA from each time point (5 μg) was reverse transcribed into Cy3-labeled cDNA using aminoallyl-modified nucleotides, as described [41]. Cy5-labeled cDNA was prepared from the reference culture using the same protocol. To minimize the variation in the measurements, the reference cDNA was pooled and aliquoted. Genomic DNA from the stationary phase culture was purified and labeled with Cy3-dUTPs as described [42]. The microarray data have been submitted to ArrayExpress (E-MEXP-894).

### Microarray analysis

Microarrays with gene-specific tags (GSTs) for the two *Sulfolobus *species were produced as previously described [43], and augmented with GSTs for additional protein-encoding genes. Probes for 2,484 and 1,946 *S. solfataricus *and *S. acidocaldarius *genes, respectively, were printed in duplicate on Ultra GAPS glass slides (Corning Life Sciences, New York, USA) with a QArray2 microarray printer (Genetix, New Milton, Hampshire, UK).

For mRNA half-life analysis, Cy5-labeled cDNA from each time point was co-hybridized for 40 h with an aliquot of Cy3-labeled reference cDNA, as described [41]. For transcript abundance analysis, 5 μg of Cy5-labeled cDNA from an untreated culture was co-hybridized with 2 μg of Cy3-labeled genomic DNA. The slides were scanned with an Agilent G2565BA microarray scanner (Agilent Technologies, Palo Alto, CA, USA), and data were collected with GenePix 5.0 software (Axon Instruments, Foster City, CA, USA). Low-quality spots were excluded as described [42]. Cy5/Cy3 log_2 _ratios of background-subtracted foreground intensities were extracted and, for each gene, averaged over the spot replicates. Only genes with at least one measurement for each time point were included. Two labeling/hybridization series were analyzed for *S. solfataricus*, and two independent cultures were analyzed for *S. acidocaldarius*.

### mRNA half-life calculations

The half-life calculations were based on two assumptions: first, that RNA decay proceeded at a constant rate over time; and second, that a given proportion (for example, 10%) of the transcripts were stable. According to the first assumption, log ratios should decrease linearly over time. Thus, we sought to normalize the log ratios for each time point (microarray) such that the decay profiles (log_2 _ratios over time), overall, were as linear as possible. This was achieved by iterating the process of: fitting a line to each decay profile by the least-squares method; and, at each time point, calculating the average (over all genes) deviation from the lines, and adjusting the log ratios at this time point with the average deviation. The process converged within five iterations. Subsequently, new lines were fitted to the adjusted decay profiles, and for each line the slope *k*, as well as the sum of the squared residuals, was calculated. To exclude noisy data, the 20% measurements that displayed the highest sum of squared residuals were excluded. To fulfil the second assumption, each *k *was normalized by subtracting the average *k *for the 10% of genes with the largest *k *values. After normalization, more than 95% of the remaining genes displayed decay profiles with a Pearson correlation coefficient of ≤ 0.95. For genes with multiple measurements, the *k *was averaged over the replicate time series. The half-life (*t*_1/2_) of a transcript was finally calculated as *t*_1/2 _= -1/*k*. As the precision in the estimates was limited for extremely long-lived transcripts, all transcripts that displayed half-lives of > 20 minutes were assigned a half-life of 20 minutes. Software for normalization is available from the authors on request.

### Quantitative real-time PCR

Primer pairs for independent confirmation of microarray data were designed against 10 *S. solfataricus *genes (SSO0071, SSO0946, SSO0708, SSO6374, SSO2652, SSO1300, SSO0446, SSO2442, SSO0277, SSO2688) selected to cover the entire range of decay rates. Primers were designed using Primer Express 2.0 (Applied Biosystems, Foster City, CA, USA) with default settings, and purchased from Operon Technologies (Cologne, Germany). Half-volume SYBR Green PCR Core Reagents (Applied Biosystems) reactions were performed using an ABI Prism 7000 Sequence Detection System (Applied Biosystems) with the following settings: 2 minutes at 50°C and 10 minutes at 95°C followed by 40 cycles of 15 s at 95°C and 1 minute at 60°C. The cDNA template was synthesized as for microarray experiments with the exception that only unmodified dNTPs were used. Three replicate reactions were performed per time point and primer pair. Microarray data-comparable log_2_expression values (M-values) for a given gene and timepoint *t *were calculated as:

M(*t *min) = CT(3 min) - CT(*t *min)

qPCR data were normalized against the transcript with the longest half-life, as for the microarray data.

### Identification of orthologous genes

We applied the Inparanoid software [44] for identifying *S. solfataricus *orthologs in *S. acidocaldarius*. Only orthologs with a one-to-one relation were considered, to avoid genes that had undergone duplication after the species diverged. The *S. acidocaldarius *genes were assigned the same COG (clusters of orthologos groups of proteins) functional category as their *S. solfataricus *orthologs.

### Half-life comparisons of functional groups of transcripts

The genes were separated into four equally sized groups according to half-life: 0 <*t*_1/2 _≤ 4, 4 <*t*_1/2 _≤ 5.5, 5.5 <*t*_1/2 _≤ 9 and *t*_1/2 _> 9. Each group was tested for over-representation of genes belonging to different functional categories in the COG database [45], relative to all genes with measured half-lives. *P *values were calculated using Fisher's exact test. To account for multiple testing, only *P *values below 0.005 were considered significant.

## Additional data files

The following additional data are included with the online version of this article. Additional data file 1 is a table with mRNA half-life data for individual genes of *S. solfataricus *and *S. acidocaldarius*. Additional data file 2 includes scatterplots of mRNA half-lives of adjacent genes in putative operons in *S. solfataricus*. Additional data file 3 includes a scatterplot displaying mRNA half-lives derived by microarray and qPCR, respectively. Additional data file 4 is a figure displaying mRNA half-life distributions for different functional categories of genes in *S. acidocaldarius*. Additional data file 5 is a figure showing mRNA half-life in relation to transcript abundance, operon length and transcript length in *S. acidocaldarius*. Additional data file 6 is a figure displaying flow cytometry data for *S. solfataricus*.

## Supplementary Material

Additional data file 1 A table with mRNA half-life data for 2,064 and 1,582 genes in *S. solfataricus *and *S. acidocaldarius*, respectively Click here for file

Additional data file 2 Scatterplots of mRNA half-life for upstream (x-axis) versus downstream (y-axis) gene in gene pairs encoded on the same strand and separated by < 10 nucleotides in *S. solfataricus* Click here for file

Additional data file 3 A scatterplot of mRNA half-lives of nine *S. solfataricus *genes, derived by qPCR (x-axis) and microarray (y-axis) analysis Click here for file

Additional data file 4 A figure displaying the distributions of *S. acidocaldarius *mRNA half-lives for different functional categories of genes in the COG database Click here for file

Additional data file 5 A figure showing mRNA half-life in relation to transcript abundance, putative operon length (in number of genes) and putative transcript length (in nucleotides) in *S. acidocaldarius* Click here for file

Additional data file 6 Flow cytometry of *S. solfataricus*, showing cell size (left) and DNA content (right) distributions of samples collected at different time points after, and immediately before (0 minutes), actinomycin D addition Click here for file
