# Supplementary material for: Global analysis of mRNA stability in the archaeon Sulfolobus
Source: Genome Biol. 2006 Oct 26;7(10):R99. doi: 10.1186/gb-2006-7-10-r99 (PMC1794556; doi:10.1186/gb-2006-7-10-r99)
Supplement: Additional data file 2 — Scatterplots of mRNA half-life for upstream (x-axis) versus downstream (y-axis) gene in gene pairs encoded on the same strand and separated by < 10 nucleotides in S. solfataricus [file gb-2006-7-10-r99-S2.pdf]

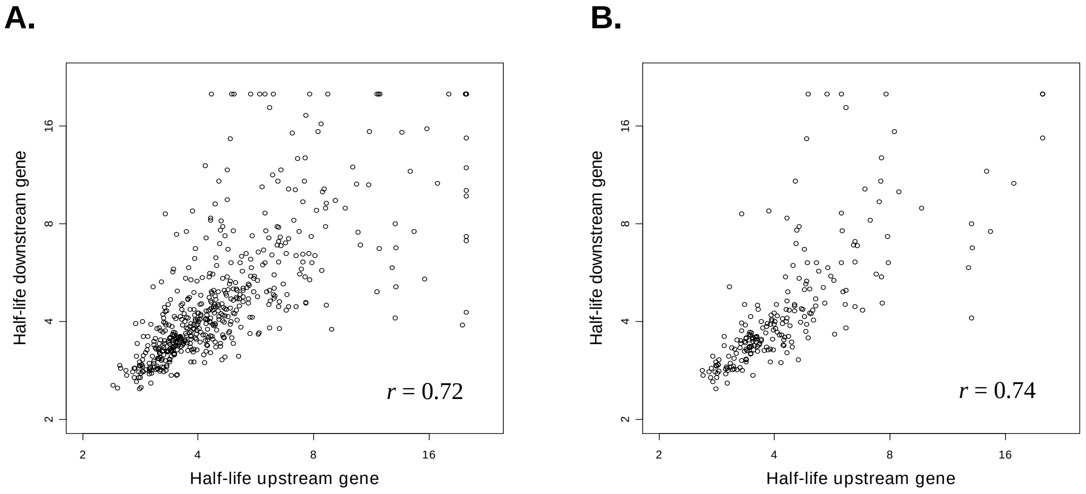

(A) Scatterplot of mRNA half-life for upstream (x-axis) vs. downstream (y-axis) gene in gene pairs encoded on the same strand and separated by <10 nt in *S. solfataricus*. (*B*) The subset of points in *A* where both genes of a pair belong to the same COG functional category, which increases the likelihood of that the genes belong to the same operon.
