# Supplementary material for: Global analysis of mRNA stability in the archaeon Sulfolobus
Source: Genome Biol. 2006 Oct 26;7(10):R99. doi: 10.1186/gb-2006-7-10-r99 (PMC1794556; doi:10.1186/gb-2006-7-10-r99)
Supplement: Additional data file 3 — A scatterplot of mRNA half-lives of nine S. solfataricus genes, derived by qPCR (x-axis) and microarray (y-axis) analysis [file gb-2006-7-10-r99-S3.pdf]

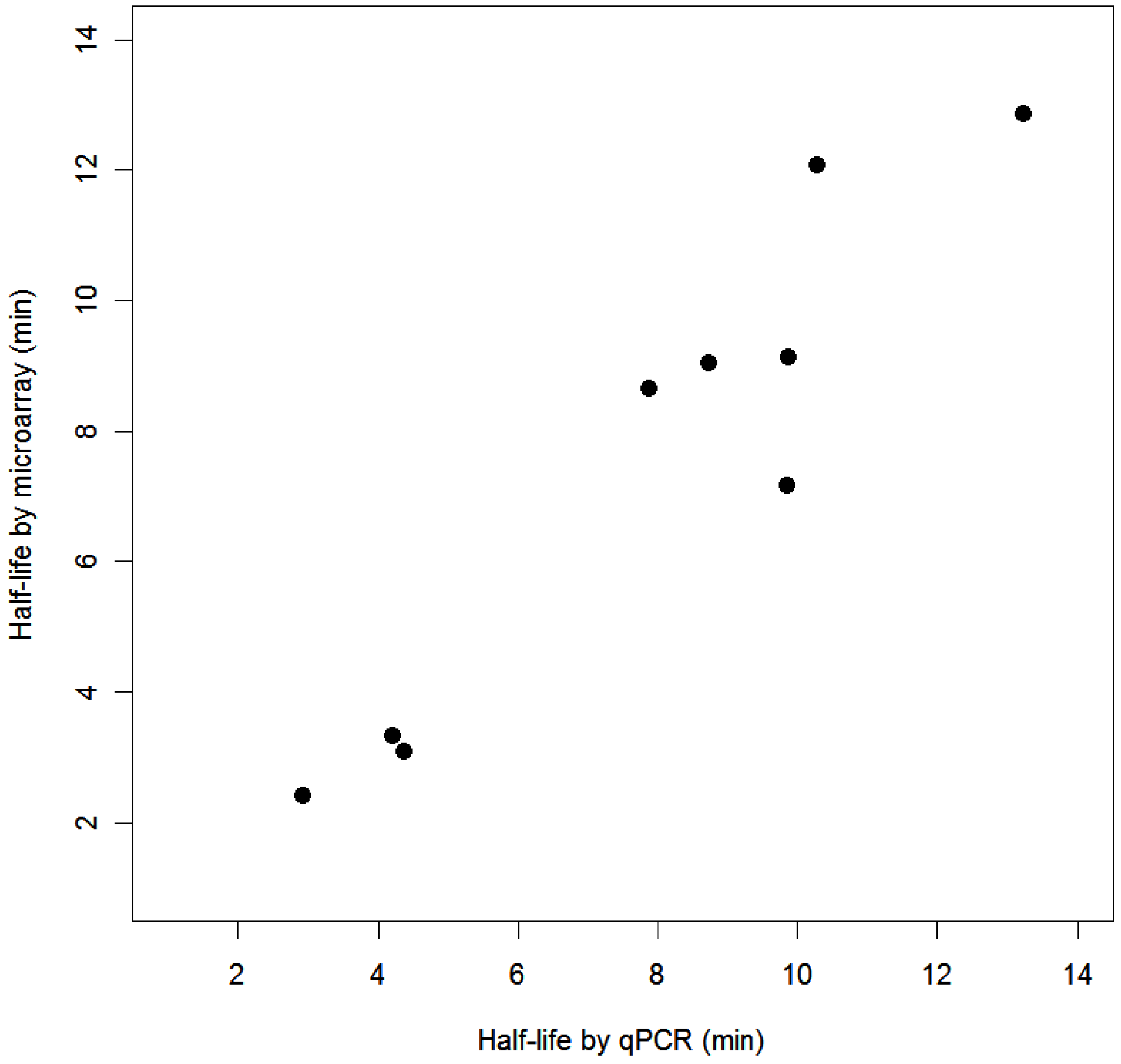


Scatterplot of mRNA half-lives for nine *S. solfataricus* genes as determined by qPCR (x-axis) and microarray (y-axis), respectively.
