# Supplementary material for: Global analysis of mRNA stability in the archaeon Sulfolobus
Source: Genome Biol. 2006 Oct 26;7(10):R99. doi: 10.1186/gb-2006-7-10-r99 (PMC1794556; doi:10.1186/gb-2006-7-10-r99)
Supplement: Additional data file 4 — A figure displaying the distributions of S. acidocaldarius mRNA half-lives for different functional categories of genes in the COG database [file gb-2006-7-10-r99-S4.pdf]

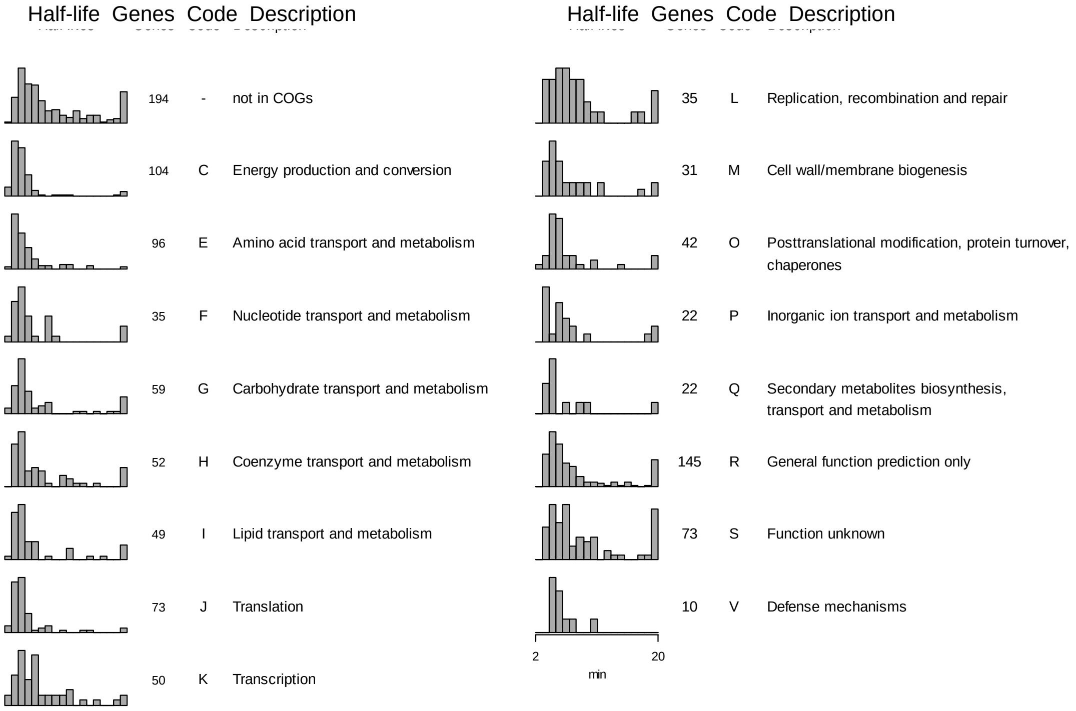

Distributions of *S. acidocaldarius* mRNA half-lives, and number of genes with estimated half-lives, for different functional categories in the COG database. Only categories with >9 genes with estimated half-lives are shown. Each bar in the histogram represents the proportion of genes with a half-life within a 1-min interval, where the first bar includes half-lives of >2 but ≤ 3 min, the second >3 but ≤ 4 min, etc. The last bar includes all transcripts with half-lives >19 min.
