# Supplementary material for: Global analysis of mRNA stability in the archaeon Sulfolobus
Source: Genome Biol. 2006 Oct 26;7(10):R99. doi: 10.1186/gb-2006-7-10-r99 (PMC1794556; doi:10.1186/gb-2006-7-10-r99)
Supplement: Additional data file 5 — A figure showing mRNA half-life in relation to transcript abundance, putative operon length (in number of genes) and putative transcript length (in nucleotides) in S. acidocaldarius [file gb-2006-7-10-r99-S5.pdf]

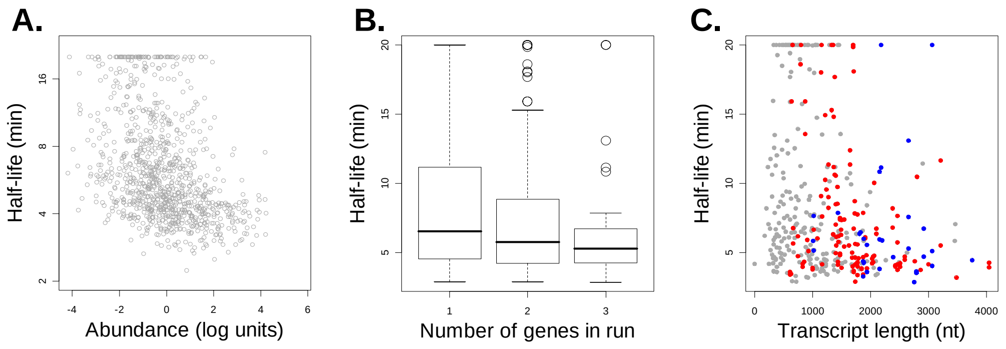

(*A*) Scatter plot of transcript abundance vs. half-life. (*B*) Box plots of half-life distributions for transcripts in one-, two- and three-gene runs. (*C*) Scatter plot of mRNA half-life vs. putative transcript length (counting from first start to last stop codon in each run). Genes belonging to one-, two and three-gene runs are shown in grey, red and blue, respectively.
