# Supplementary figures and images for: Global analysis of mRNA stability in the archaeon Sulfolobus
Source: Genome Biol. 2006 Oct 26;7(10):R99. doi: 10.1186/gb-2006-7-10-r99 (PMC1794556; doi:10.1186/gb-2006-7-10-r99)

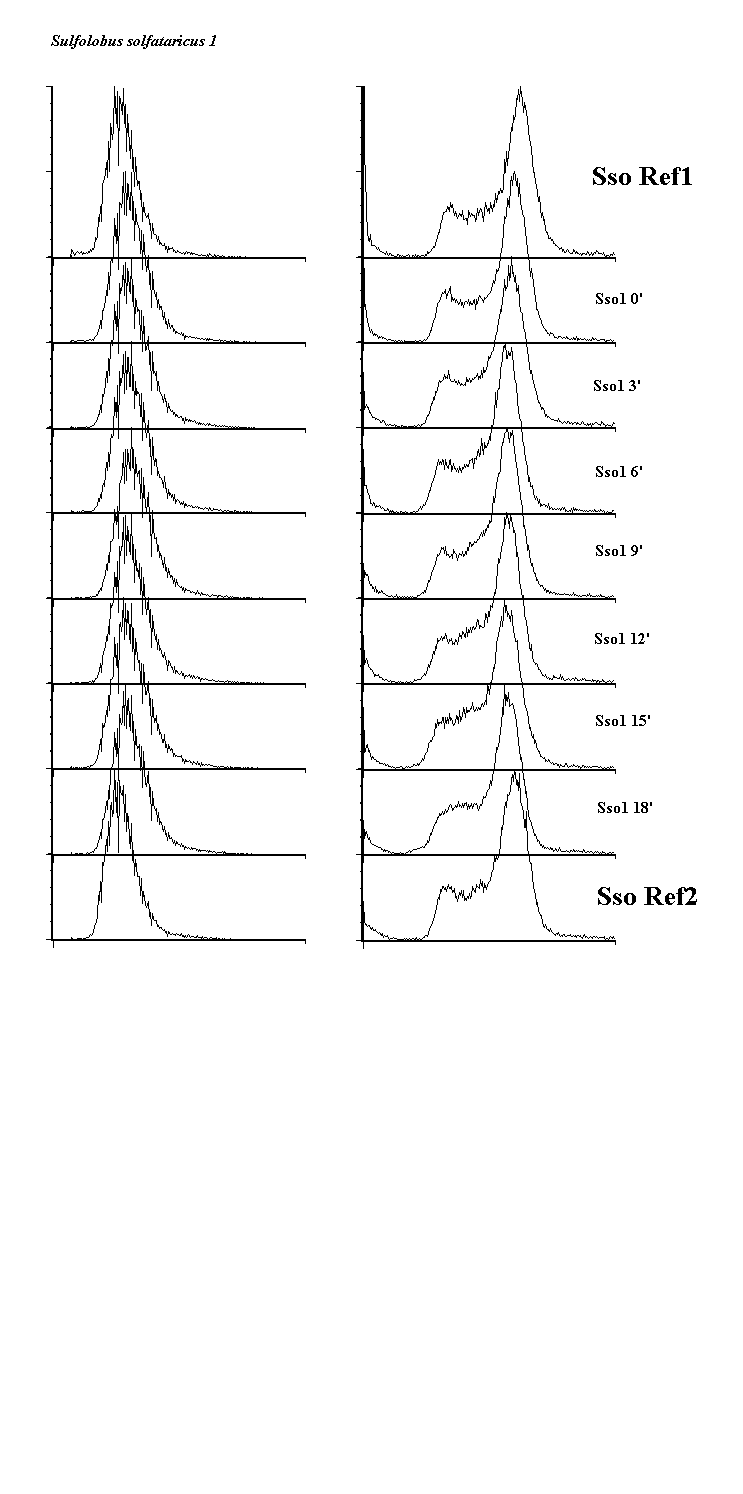

Supplement: Additional data file 6 — Flow cytometry of S. solfataricus, showing cell size (left) and DNA content (right) distributions of samples collected at different time points after, and immediately before (0 minutes), actinomycin D addition [file gb-2006-7-10-r99-S6.bmp]
